# HBV Viral Load and Liver Enzyme Levels May Be Associated with the Wild* MBL2* AA Genotype

**DOI:** 10.1155/2017/3718451

**Published:** 2017-03-12

**Authors:** Tuane Carolina Ferreira Moura, Ednelza da Silva Graça Amoras, Mauro Sérgio Araújo, Maria Alice Freitas Queiroz, Simone Regina Souza da Silva Conde, Sâmia Demachki, Rosimar Neris Martins-Feitosa, Luiz Fernando Almeida Machado, Izaura Maria Vieira Cayres-Vallinoto, Ricardo Ishak, Antonio Carlos Rosário Vallinoto

**Affiliations:** ^1^Laboratory of Virology, Institute of Biological Sciences, Universidade Federal do Pará (UFPA), Guamá, 66075-110 Belém, PA, Brazil; ^2^João de Barros Barreto University Hospital, Federal University of Pará (Universidade Federal do Pará (UFPA)), Guamá, 66073-000 Belém, PA, Brazil; ^3^School of Medicine, Institute of Health Sciences, Universidade Federal do Pará (UFPA), Umarizal, 66055-380 Belém, PA, Brazil; ^4^Hepatology Outpatient Service, Santa Casa de Misericórdia do Pará, Umarizal, 66055-380 Belém, PA, Brazil

## Abstract

The present study investigated the frequencies of rs1800450 (*MBL* ^⁎^B, G>A), rs1800451 (*MBL* ^⁎^C, G>A), and rs5030737 (*MBL* ^⁎^D, C>T) polymorphisms in exon 1 of the* MBL2* gene among patients with chronic viral hepatitis. Blood samples from patients infected with hepatitis B virus (HBV; *n* = 65), hepatitis C virus (HCV; *n* = 92), and a noninfected control group (*n* = 300) were investigated. The presence of polymorphisms was detected using a real-time polymerase chain reaction to correlate with liver disease pathogenesis and fibrosis staging according to the Metavir classification. The genotypic and allelic frequencies showed no significant differences between the groups, but patients with active HBV and the wild* AA* genotype presented a positive correlation between increased transaminase and HBV DNA levels and the presence of mild to moderate fibrosis. Patients with HCV and the wild* AA* genotype presented mild inflammation and higher HCV RNA levels, although the same association was not observed for the fibrosis scores. The results suggest that the mutations in exon 1 of the* MBL2* gene do not contribute directly to the clinical and laboratory features of HCV and HBV infections, but further studies should be performed to confirm whether the wild* AA* genotype has indirect effect on disease progression.

## 1. Introduction

Mannose-binding lectin (MBL) is synthesized by hepatocytes and belongs to the collectin family [1]. MBL plays a key role in the innate immune response, with a specialized capacity to recognize several carbohydrate (mannose, glucose, and N-acetyl-glucosamine) components existent on the surface of microorganisms but present in small amounts or not exposed in the human cell [2]. This interaction leads to activation of the complement system and promotes the elimination of the pathogen through the formation of the membrane attack complex (MAC). In addition, MBL can also function as an opsonin to facilitate phagocytosis of bacteria, virus, and fungi [3].

The deficiency in MBL production may be associated with greater susceptibility to infectious and autoimmune diseases, which affect the severity and progression of the clinical condition [4]. MBL shows clinical relevance when the adaptive immunity is already compromised as a result of immune immaturity, comorbidities, specific drug therapies, cystic fibrosis [5], chemotherapy [6], and in transplanted patients [7]. MBL has been reported to play a role in the regulation and production of inflammatory cytokines [interleukin- (IL-) 6, IL-1, and tumor necrosis factor- (TNF-) *α*], which highlights its influence on the severity and progression of infections [8].

Three single nucleotide polymorphisms (SNP) in exon 1 of the* MBL2 *gene are located in codons 52,* MBL*^*∗*^*D* (rs5030737 – C>T); 54,* MBL*^*∗*^*B* (rs1800450 - G>A); and 57,* MBL*^*∗*^*C* (rs1800451 - G>A). They are collectively known as* MBL*^*∗*^*O,* whereas the wild-type allele is named* MBL*^*∗*^*A *[9]. The SNP in codon 52 (**C**GT/**T**GT) deregulate oligomer formation due to the generation of disulfide bridges [10]. Changes in codons 54 (G**G**C/G**A**C) and 57 (G**G**A/G**A**A) interrupt the Gly-X-Y repeat present in the collagen-like region of this collectin. This has an impact on the helix and MBL structure, which generates defective interactions with MBL-associated serine proteases (MASPs), the Carbohydrate Recognition Domain and its target. [11]. As a consequence, a possible incorrect interaction of MASPs during C4 and C2 cleavage (products required for the formation of the C3 convertase) leads to the equivocal activation of the complement system, resulting in low circulating MBL levels [11, 12].

There are differences between serum MBL levels according to genotypes* AA, AO, *and* OO*, and the frequencies of these genotypes are quite variable in different ethnic groups [13]. For instance, the allele* MBL*^*∗*^*B* was found to be more frequent in European and Asian populations [14], and the allele ^*∗*^*C *was more frequent in sub-Saharan populations [15].

Hepatitis B virus (HBV) and hepatitis C virus (HCV) are the main causes of chronic liver disease worldwide and are responsible for the most severe forms of liver cirrhosis and hepatocellular carcinoma (HCC) [16]. The role of MBL in the development of cirrhosis is related to increased hepatotoxic damage, and its origin is attributed to viral infection and/or immunodeficiency associated with* MBL2* alleles that affect liver pathology [17, 18].* MBL2* gene mutations have been related to the progression of the infection to the chronic phase, with the development of cirrhosis and hepatocellular carcinoma, and to the different responses to therapy [19, 20]. Recently, it was reported that the presence of mutant alleles in the promoter region (−221 C>G) of the MBL2 gene could protect against the more severe forms of the liver injury (cirrhosis and HCC) [21], and a better liver function was shown as associated with high MBL serum levels [22]. Furthermore, Zupin et al. [23] reported that MBL2 polymorphisms were not associated with HCV infection susceptibility and with spontaneous viral clearance, while* MBL2*^*∗*^*O* allele,* OO* genotype, and* HYO* haplotype, all correlated with low or deficient MBL expression, were associated with sustained virological response (SVR).

The present study investigated the frequencies of* MBL2* gene polymorphisms* MBL*^*∗*^*D* (rs5030737, C>T),* MBL*^*∗*^*B* (rs1800450, G>A), and* MBL*^*∗*^*C* (rs1800451, G>A) in patients with chronic hepatitis B and C viral infections and the correlation with laboratory markers of inflammation and structural changes in the liver parenchyma.

## 2. Materials and Methods

### 2.1. Study Population

This was a cross-sectional study which involved a total of 157 consecutive patients with HBV (*n* = 65) and HCV (*n* = 92) attending the hepatology outpatient service of the Holy House of Mercy Foundation of Pará (Fundação Santa Casa de Misericórdia do Pará (FSCMPA)) and the João de Barros Barreto University Hospital.

All the patients were clinically evaluated and underwent biochemical [ALT (alanine aminotransferase), AST (aspartate aminotransferase), and GGT (gamma-glutamyl transferase)], serological (HBsAg (HBV surface antigen), HBeAg (HBV e antigen), anti-HBeAg, anti-HBc (IgM and IgG against HBcAg), and anti-HCV], and virological (HBV DNA, HCV RNA, and genotyping) tests, ultrasound, endoscopy, and liver biopsy. The laboratory information was obtained from their clinical records at the time of entry in the study. Additionally, HBV infected subjects were classified as active (HBV-AC) and inactive (HBV-IC) carriers according to serological and virological markers.

The criteria for inclusion in the study comprised of persons who were 18 years of age or older, both sexes, HBsAg carriers for more than 6 months, positive HCV RNA, and patients with or without high ALT, AST, and GGT values. Individuals who did not meet the above-mentioned requirements, those coinfected with hepatitis D virus (HDV), those with human immunodeficiency virus type 1 (HIV-1), and patients who had used or were using specific antiviral therapies against HBV or HCV were excluded.

This study was approved by the Research Ethics Committee of the Holy House of Mercy Foundation of Pará (protocol #772.782/2014) and the João de Barros Barreto University Hospital (protocol 962.537/2015). All persons who agreed to participate in the study signed an informed consent form.

### 2.2. Biological Samples

Blood samples were collected from the patients with the aid of vacuum tubes containing ethylenediaminetetraacetic acid (EDTA) as an anticoagulant. Peripheral blood cells and plasma were obtained after centrifugation and stored at −20°C prior to the analysis.

### 2.3. DNA Extraction

Total DNA was extracted from peripheral blood cells from the study samples according to a previously described protocol [24]. The procedure included the following steps: cell lysis, protein precipitation, DNA precipitation, and DNA hydration.

### 2.4. Assessment of MBL2 Gene Polymorphisms

The polymorphisms rs1800450 (G>A), rs5030737 (C>T), and rs1800451 (G>A) of exon 1 in the* MBL2* gene were genotyped by real-time polymerase chain reaction (RT-PCR) in a StepOne PLUS Sequence Detector (Applied Biosystems, Foster City, CA, USA). The assays performed for each polymorphism contained a pair of primers and a probe labelled with VIC and FAM for each allele of the respective polymorphisms. Assays C__12336609_20, C__2336610_20, and C__2336608_20, which were predesigned by Applied Biosystems, were used for polymorphisms rs1800450 (G>A), rs5030737 (C>T), and rs1800451 (G>A), respectively. Each reaction contained 10 *µ*L of TaqMan® Universal PCR Master Mix [2x], 1 *µ*L of TaqMan Assay Buffer [20x], 6 *µ*L of water, and 20 ng of DNA in a final reaction volume of 20 *µ*L. The following temperature cycles were used for the amplification and detection of alleles: 60°C for 30 seconds, followed by 95°C for 10 minutes and 50 cycles of 92°C for 30 seconds and 60°C for 1 minute and 30 seconds.

### 2.5. Statistical Analysis

The allelic and genotypic frequencies were obtained by direct counting. The Hardy-Weinberg equilibrium was evaluated using the Chi-square test (*χ*^2^). Comparative analyses of the allelic and genotypic frequencies were performed using the *G*-test and Fisher's exact test. Comparisons between the enzyme levels (AST, ALT, and GGT) and HBV and HCV viral load levels were performed using the Mann–Whitney and Pearson tests. Calculations and graphical plots were performed using the BioEstat 5.0 [25] and GraphPad Prism 5.0 [26] software. Significance level was set at 5% (*p* value ≤ 0.05).

## 3. Results

The mean plasma concentrations of ALT and AST were higher, but without significance, in the groups of patients with HCV (77.64 and 65.35 UI/L, respectively) and HBV-AC (67.2 and 67.05 UI/L, respectively) as compared with HBV-IC (27.10 and 26.88 UI/L). The highest GGT level was observed in the group of patients with HCV (96.87 UI/L); no difference was observed between HBV-AC and HBV-IC (56.69 and 58.75 UI/L, respectively).

The stage of fibrosis scores varied from F0 to F4 among patients with HCV, with 59% presenting mild and moderate fibrosis (F0–F2). Among those with HBV, 100% of the inactive carriers presented mild to moderate fibrosis (F0-F1) and 48.5% of the active carriers had severe fibrosis and cirrhosis (F3-F4).

Table 1 shows that 81% and 100% of the HBV-AC and HBV-IC, respectively, presented mild inflammation (A0-A1), whereas 40.8% of the patients with HCV showed moderate inflammatory activity (A2).

The* AA* genotype and ^*∗*^*A* allele were the most frequently found in all groups. The* OO* genotype (alleles ^*∗*^*B*, ^*∗*^*C*, and ^*∗*^*D*) had similar frequencies among the groups. The* BB, CC*, and* DD* genotypes were not found. No significant differences were observed in the genotypic frequencies of the exon 1 polymorphisms of the* MBL2* gene (Table 2).

The correlation between the polymorphisms and the degree of inflammatory response showed that the* AA* genotype was the most frequent in patients with HBV and HCV in both mild and severe inflammatory disease. The same profile was observed for the fibrosis scores, but no significant results were obtained compared to the analyzed groups (Table 3).

**Figure 1:**
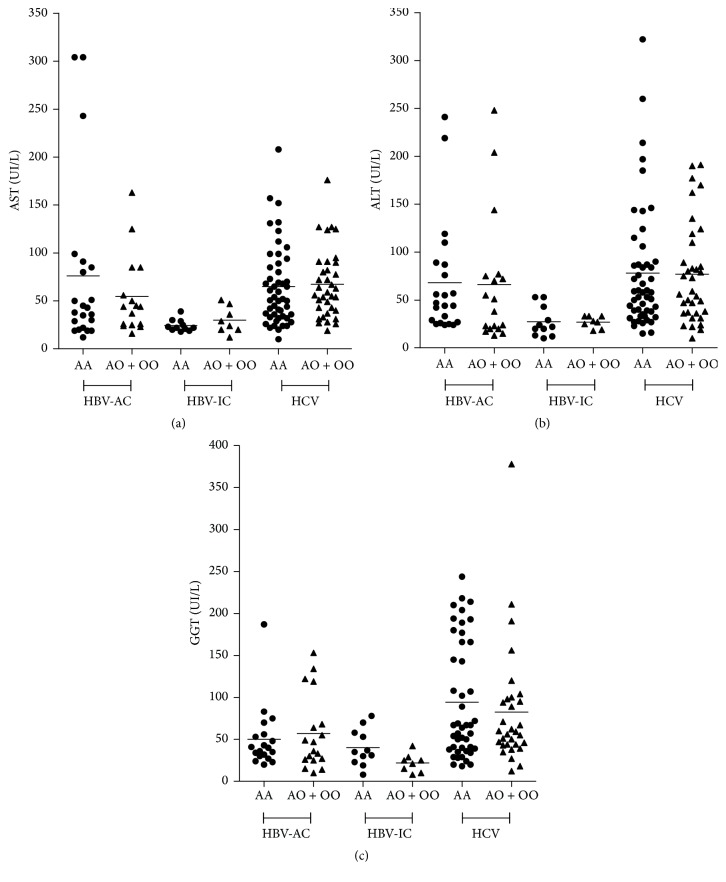
Plasma levels of liver enzymes according to the genotype of the* MBL2* gene polymorphisms in exon 1 (rs1800450, rs1800451, and rs5030737). AST (a), ALT (b), and GGT (c) levels in patients with active hepatitis B (HBV-AC), inactive hepatitis B (HBV-IC), and hepatitis C (HCV) infections. AST: aspartate aminotransferase; ALT: alanine aminotransferase; and GGT: gamma-glutamyl transferase. HBV: hepatitis B virus and HCV: hepatitis C virus. Reference values: AST (16–40 IU/L), ALT (08–54 IU/L), and GGT (08–63 IU/L). Mann–Whitney test (*p* < 0.05).

Biochemical markers were compared between the HBV-AC and HBV-IC group and in the group of patients with HCV. No significant differences were observed between the presence of polymorphisms (*AA* versus* AO/OO*) and the plasma levels of the liver enzymes AST, ALT, and GGT (Figures 1(a), 1(b), and 1(c)).

**Figure 2:**
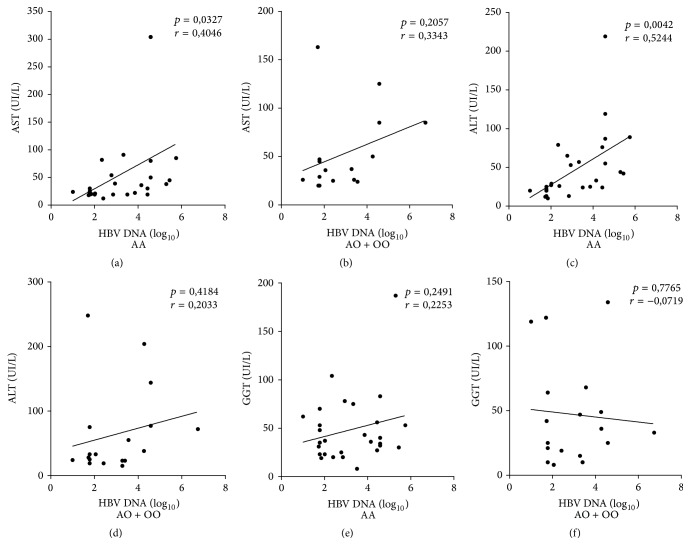
Pearson's correlation analysis of the liver enzymes levels according to the HBV DNA viral load (log_10_) and the* AA* and* AO/OO MBL2 *gene genotypes. ((a), (b)) AST: aspartate aminotransferase; ((c), (d)) ALT: alanine aminotransferase; and ((e), (f)) GGT: gamma-glutamyl transferase (*p* < 0.05).

**Figure 3:**
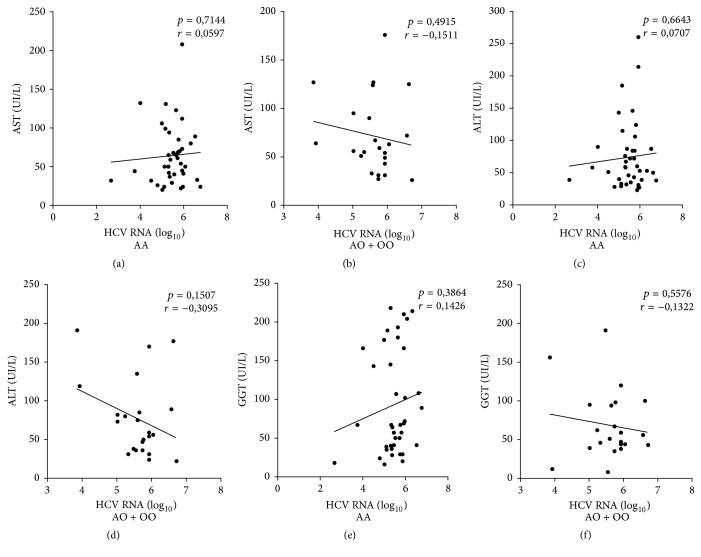
Pearson's correlation analysis of the liver enzymes levels according to the HBV RNA viral load (log_10_) and the* AA* and* AO/OO MBL2* gene genotypes. ((a), (b)) AST: aspartate aminotransferase; ((c), (d)) ALT: alanine aminotransferase; and ((e), (f)) GGT: gamma-glutamyl transferase (*p* < 0.05).

The comparison of AST, ALT, and GGT levels in relation to HBV DNA viral load (log_10_) according to the genotype showed a positive correlation between an increased viral load and increased liver enzyme levels in the group with the* AA *genotype, with a significance difference observed only for AST and ALT (Figures 2(a) and 2(c)). But, regarding the HCV RNA viral load (log_10_), no statistical significance was reported (Figure 3).

**Figure 4:**
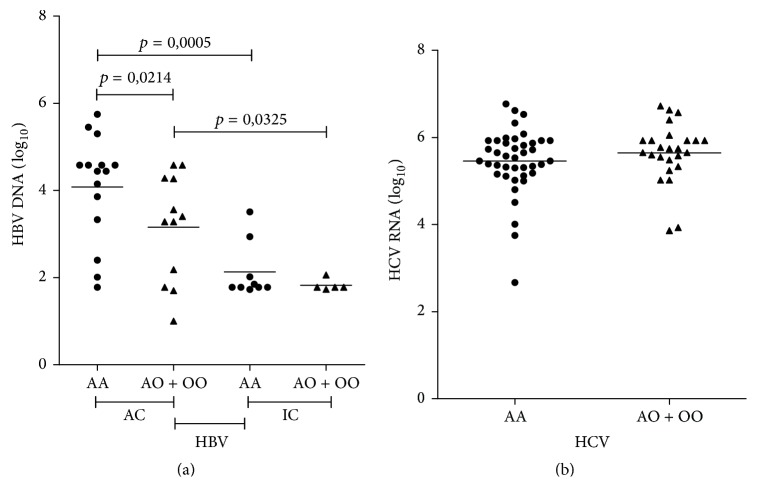
Viral load according to the different genotypes of the mannose-binding lectin gene polymorphisms in exon 1 (rs1800450, rs1800451, and rs5030737) in (a) HBV-AC × IC HBV DNA and (b) HCV RNA. HBV: hepatitis B virus and HCV: hepatitis C virus. Mann–Whitney test (*p* < 0.05). (AC: active carriers and IC: inactive carriers).

Among the patients with HBV-AC and HBV-IC (Figure 4(a)), there was a significant association of a higher HBV DNA load (log_10_) with the* AA *genotype as compared with carriers of* AO/OO* genotypes, but this association was significant only in the group HBV-AC. There were no significant differences in the HCV RNA levels (log_10_) according to the genotypes (Figure 4(b)).

**Figure 5:**
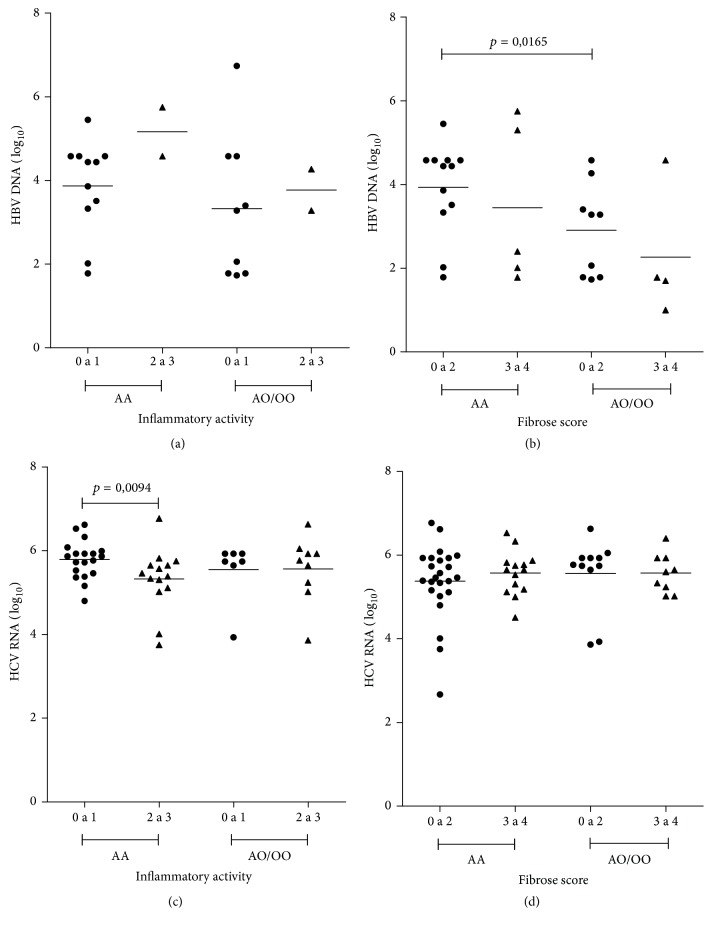
Viral load according to the different genotypes of the mannose-binding lectin gene. (a): HBV DNA × inflammatory activity, (b): HBV DNA × fibrosis score, (c): HCV RNA × inflammatory activity, and (d): HCV RNA × fibrosis score. HBV: hepatitis B virus and HCV: hepatitis C virus. Inflammatory activity (0 to 1: no inflammation and mild levels and 2 to 3: moderate and high levels) and fibrosis score (0 to 2: mild and moderate and 3 to 4: severe and cirrhosis) Metavir. Mann–Whitney test (*p* < 0.05).

The relationship between the viral load and inflammatory activity, among HBV infected persons, showed no significant difference in relation to the genotype (Figure 5(a)). However, the viral load was higher among the patients with the* AA* genotype (F0–F2) compared with HBV carriers of* AO/OO* genotypes (F0–F2) (Figure 5(b)).

The inflammatory activity of HCV, according to the genotypes, showed that persons with the* AA* genotype presented significantly higher RNA levels associated with mild inflammation (A0-A1), but, patients with the* AO/OO* genotypes presented similar HCV RNA levels regardless of the degree of liver inflammation (Figure 5(c)). HCV RNA levels were similar as compared to the fibrosis scores (Metavir), regardless of the genotypes analyzed (Figure 5(d)).

## 4. Discussion

Genetic polymorphisms are capable of influencing the immune response during inflammation and tissue damage [27]. Our observation that the wild-type* AA* genotype was present in the highest frequencies in both the patients and controls suggests the lack of a direct modulation of infection with SNPs with the infection, which corroborated previous results obtained with HCV carriers [28]. It is possible that the lack of association of MBL is a consequence of its role as an acute phase protein, which is not relevant when dealing with patients who are in the chronic phase of the disease.

The combination of the mutant genotype* OO* (genotypes* BC, DB, *and* CD*) was distributed in a similar frequency among HBV and HCV infected persons and the control group. There was no association with the progression of chronic liver disease as the same genotypic and allelic profiles were observed regardless of the inflammation and liver fibrosis levels. Nevertheless, a greater frequency of genotype* AA* was observed in patients with mild and moderate fibrosis in all groups. These results indicate that, in the population studied, these polymorphisms did not influence the progression to one of the most severe forms of the disease (i.e., cirrhosis), corroborating other studies that also found no correlation between these polymorphisms and chronic liver disease progression [28–31]. It is relevant to mention that some studies found an association between the progression of active chronic liver disease or cirrhosis with the* MBL*^*∗*^*B* allele and the MBL/MASP-1 complex in patients with HCV [20, 32] and HBV [33–35].

The absence of association between ALT, AST, and GGT levels with the different genotypes in both viral infections may indicate that the polymorphisms did not directly affect the levels of these enzymes during HBV and HCV infections, but also, it could be related to the known frequent variations which occur in the levels and activity of transaminases during viral liver infection [36].

The presence of* AO* and* OO* genotypes is frequently related to changes in the MBL structure [11, 37], which would lead to defective antiviral activity, thereby preventing the destruction of infected hepatocytes. This situation would lead to increased HBV DNA and HCV RNA levels and influencing the severity and clinical progression of liver disease, with the increase in transaminase activity [38]. However, this scenario was not observed in our analysis relating both HBV and HCV viral load and transaminases with this polymorphism. In contrast, we observed that patients with the wild-type* AA* genotype presented a positive correlation between increased AST and ALT levels and the HBV DNA levels (i.e., the higher the viral load in the liver of these patients, the higher the levels of these biochemical markers of liver injury), but not with HCV RNA levels.

The highest values of viral loads were found among HBV carriers with the wild-type* AA* genotype and mild to moderate fibrosis (F1-F2), suggesting a possible increase in liver damage repair mechanisms in the initial phase [39] and that a large number of hepatocytes could still be infected. This profile differs from that observed in cases of severe fibrosis and cirrhosis, where the presence of infected cells is decreased by the replacement of the liver parenchyma with fibrotic tissue, leading to a decrease in the viral load.

Patients with the wild* AA* genotype who presented mild inflammatory activity showed the highest HCV RNA levels, but the same finding was not observed for the fibrosis scores. These results suggest the fluctuating nature of the liver inflammatory process, with periods of worsening and improvement. The nature of the liver inflammatory process is as follows: with periods of remissions, fibrosis is a mechanism that, at first, repairs the damage with the goal of limiting the extension of the inflammatory process. However, as a result of the persistence of the infection and continuing damage to the liver, cirrhosis develops [40] and common final stage of the hepatic pathological processes [41].

It is relevant to mention that there are no previous published attempts to associate the levels of liver enzymes (AST, ALT, and GGT) and viral load (HBV DNA and HCV RNA) with the polymorphisms present in exon 1 of the* MBL2* gene. Therefore, the unprecedented results presented demonstrate the importance of understanding the role of* MBL2* gene polymorphisms in the context of the progression of chronic viral liver infection.

## 5. Conclusion

The present study suggests that the mutations in exon 1 of the* MBL2* gene (in homo or heterozygosis) do not contribute directly to the clinical and laboratory features of HCV and HBV infections. Further studies should be performed to confirm whether the wild* AA* genotype has indirect effect on disease progression.

## Figures and Tables

**Table 1:** Clinical, biochemical, and histopathological data according to the Metavir scores of the study population.

Variables	HBV		HCV
	AC	IC	
*Sex*			
Male *n* (%)	22 (55.0%)	19 (76.0%)	50 (54.4%)
Female *n* (%)	18 (45.0%)	06 (24.0%)	42 (45.6%)
*Mean age (years ± SD)*			
Male	51.90 ± 14.20	52.58 ± 15.49	53.60 ± 11.18
Female	42.90 ± 13.49	42.66 ± 06.88	54.29 ± 08.34
*Liver enzymes*			
ALT (UI/L) Mean ± SD (08–54 UI/L)	67.20 ± 62.34	27.10 ± 12.27	77.64 ± 59.27
AST (UI/L) Mean ± SD (16–40 UI/L)	67.05 ± 72.48	26.88 ± 10.13	65.35 ± 39.27
GGT (UI/L) Mean ± SD (08–63 UI/L)	56.69 ± 92.12	58.75 ± 80.98	96.87 ± 90.76
*Fibrosis scores* ^a^			
F 0 to 2 *n* (%)	17 (51.5)	07 (100.0)	36 (59.0%)
F 3 to 4 *n* (%)	16 (48.5)	00 (00.0)	25 (41.0%)
*Inflammatory activity* ^b^			
A 0 to 1 *n* (%)	16 (80.0)	07 (100.0)	42 (59.2%)
A 2 to 3 *n* (%)	04 (20.0)	00 (00.0)	29 (40.8%)

HBV: hepatitis B virus (AC: active carrier; IC: inactive carrier) and HCV: hepatitis C virus. ALT: alanine aminotransferase; AST: aspartate aminotransferase; and GGT: gamma-glutamyl transferase. ^a^Fibrosis score (0 to 2: mild and moderate and 3 to 4: severe and cirrhosis) Metavir. ^b^Degree of inflammation (0 to 1: mild inflammation and 2 to 3: severe inflammation).

**Table 2:** Distribution of genotypic and allelic frequencies of polymorphisms of exon 1 of the *MBL2 *gene (rs1800450, rs1800451, and rs5030737) in the samples from patients infected with HBV and HCV and control samples.

Genetic profile	HBV	HCV	Control	*p*
	*n* = 65	*n* = 92	*n* = 300	
	*n* (%)	*n* (%)	*n* (%)	
Genotypes				
*AA*	37 (56.9)	52 (56.5)	167 (55.7)	^a^0.9942
*AO*	25 (38.5)	36 (39.1)	123 (41.0)	^b^0.8474
*AB*	16 (24.6)	28 (30.4)	91 (30.3)	^c^0.8763
*AC*	07 (10.8)	08 (08.7)	18 (06.0)	
*AD*	02 (03.1)	—	14 (04.7)	
*OO*	03 (04.6)	04 (04.4)	10 (03.3)	
*BB*	—	—	—	
*BC*	02 (03.1)	03 (03.3)	05 (01.7)	
*BD*	01 (01.5)	01 (01.1)	04 (01.3)	
*CC*	—	—	—	
*CD*	—	—	01 (00.3)	
*DD*	—	—	—	
Alleles				
^*∗*^ *A*	0.762	0.761	0.762	^a^0.9891
^*∗*^ *O*	0.238	0.239	0.238	^b^0.9975
^*∗*^*B*	0.146	0.174	0.166	^c^0.9823
^*∗*^*C*	0.069	0.060	0.040	
^*∗*^*D*	0.023	0.005	0.032	

*G*-test, genotypes and Fisher's exact test, alleles. HBV: hepatitis B virus and HCV: hepatitis C virus. ^a^HBV versus HCV; ^b^HBV versus Control; and ^c^HCV versus Control.

**Table 3:** Correlation between polymorphisms in exon 1 of the *MBL2* gene with the inflammatory activity and fibrosis score in samples from patients infected with HBV and HCV.

Genetic profile infection	Inflammatory activity			Fibrosis score		
	0 to 1	2 to 3	*p*	0 to 2	3 to 4	*p*
	*n* (%)	*n* (%)		*n* (%)	*n* (%)	
HBV						

*AA*	12 (52.2)	02 (50.0)	^†^0.4286	13 (54.2)	09 (56.2)	^†^0.3424
*AO*	10 (43.5)	01 (25.0)		09 (37.5)	07 (43.8)	
*OO*	01 (04.3)	01 (25.0)		02 (08.3)	00 (00.0)	

^*∗*^*A*	0.739	0.625	^*∗*^0.3896	0.729	0.781	^*∗*^0.3996
^*∗*^*O*	0.261	0.375		0.271	0.219	

*AA*	12 (52.2)	02 (50.0)	^*∗*^0.6733	13 (54.2)	09 (56.2)	^*∗*^0.5778
*AO + OO*	11 (47.8)	02 (50.0)		11 (55.8)	07 (53.8)	

HCV						

*AA*	23 (63.9)	15 (60.0)	^†^0.2833	26 (61.9)	17 (58.6)	^†^0.2966
*AO*	11 (30.5)	10 (40.0)		14 (33.3)	12 (41.4)	
*OO*	02 (05.6)	00 (00.0)		02 (04.8)	00 (00.0)	

^*∗*^*A*	0.792	0.800	^*∗*^0.5432	0.786	0.793	^*∗*^0.5439
^*∗*^*O*	0.208	0.200		0.214	0.207	

*AA*	23 (63.9)	15 (60.0)	^*∗*^0.4826	26 (61.9)	17 (58.6)	^*∗*^0.4862
*AO + OO*	13 (36.1)	10 (40.0)		16 (38.1)	12 (41.4)	

^†^ *G*-test and ^*∗*^Fisher's exact test. HBV: hepatitis B virus and HCV: hepatitis C virus. Inflammatory activity (0 to 1: no inflammation and mild levels and 2 to 3: moderate to high levels); fibrosis score (0 to 2: mild and moderate and 3 to 4: severe and cirrhosis).
